# Efficacy of remote ischemic conditioning on improving WMHs and cognition in very elderly patients with intracranial atherosclerotic stenosis

**DOI:** 10.18632/aging.101764

**Published:** 2019-01-28

**Authors:** Da Zhou, Jiayue Ding, Jingyuan Ya, Liqun Pan, Chaobo Bai, Jingwei Guan, Zhongao Wang, Kexin Jin, Qi Yang, Xunming Ji, Ran Meng

**Affiliations:** ^1^Department of Neurology, Xuanwu Hospital, Capital Medical University, Beijing, China; ^2^Department of Neurosurgery, Xuanwu Hospital, Capital Medical University, Beijing, China; ^3^Department of Radiology, Xuanwu Hospital, Capital Medical University, Beijing, China; ^4^Advanced Center of Stroke, Beijing Institute for Brain Disorders, Beijing, China; ^5^Department of China-America Institute of Neuroscience, Xuanwu Hospital, Capital Medical University, Beijing, China; ^*^Equal contribution

**Keywords:** remote ischemic conditioning, octo- and nonagenarians, intracranial atherosclerotic stenosis, white matter hyperintensities, cognitive impairment

## Abstract

Our previous study revealed that remote ischemic conditioning (RIC) reduced the incidence of stroke or TIA in octo- and nonagenarians with intracranial atherosclerotic stenosis (ICAS). Herein, we aimed to investigate whether RIC would influence the progression of white matter hyperintensities (WMHs) and cognitive impairment in the same group of patients. Fifty-eight patients with ICAS were randomly assigned in a 1:1 ratio to receive standard medical treatment with RIC (n=30) versus sham-RIC (n=28). The RIC protocol consisted of 5 cycles of alternating 5-min ischemia and 5-min reperfusion applied in the bilateral upper arms twice daily for 300 days. The efficacy outcomes included WMHs change on T2 FLAIR sequences, estimated by the Fazekas scale and Scheltens scale, cognitive change as assessed by the MMSE and MoCA, and some clinical symptoms within 300-day follow-up. Compared with the baseline, RIC treatment significantly reduced Fazekas and Scheltens scores at both 180-day (both *p*<0.05) and 300-day (both *p*<0.01) follow-ups, whereas no such reduction was observed in the control group. In the RIC group, Fazekas scores were significantly lower at 300-day follow-up (*p*<0.001) while Scheltens scores were significantly lower at both 180-day and 300-day follow-ups (both *p*<0.001), as compared with the control group. There were statistically significant between-group differences in the overall MMSE or MoCA scores, favoring RIC at 180-day and 300-day follow-ups (all *p*<0.05). RIC may serve as a promising adjunctive to standard medical therapy for preventing the progression of WMHs and ameliorating cognitive impairment in very elderly patients with ICAS.

## INTRODUCTION

White matter hyperintensities (WMHs), also known as leukoaraiosis, are not uncommon neuroimaging findings in elderly individuals, particularly in those with known vascular risk factors and associated cerebrovascular diseases [1, 2]. Anatomically, blood vessels supplying the white matter (WM) are mainly long, penetrating arterioles with few anatomic branches, and the WM receives less blood flow than that in the gray matter. These vascular features may render the WM more vulnerable to ischemic insult. In addition, a group of aging-related vascular changes, such as increased tortuous arterioles, thickened vascular walls due to collagen deposition, decreased vascular density and impaired cerebral blood flow (CBF) autoregulation could further exacerbate WM damage [3, 4]. Clinically, a high WMH burden is considered to be associated with progressive cognitive impairment, increased risk of stroke and dementia, gait disturbance, depression and even death [2, 5, 6].

Although the pathophysiological mechanisms underlying WMHs remain unclear, endotheial dysfunction and chronic hypoperfusion secondary to atherosclerosis appear to play a central role in the initiation and progression of WMHs [7, 8]. Recently, intracranial atherosclerotic stenosis (ICAS) has been suggested as a major cause of chronic cerebral ischemia (CCI) worldwide, notably in Asians [9, 10]. ICAS-induced CCI is the basis for vascular cognitive impairment and can promote the occurrence and recurrence of stroke. In addition, a growing body of evidence indicates a strong association between ICAS and WMHs in stroke patients as well as in an asymptomatic population [7, 8, 11, 12]. Severe stenosis or occlusion of intracranial arteries can cause diffuse cerebral hypoperfusion, and if this CBF reduction is continuous, WMHs may develop, followed by stroke and impaired cognition. Other possible explanations for a high prevalence of WMHs in ICAS patients include artery-to-artery embolism from atherosclerotic lesions and shared risk factors by ICAS and WMHs [7, 8].

To date, despite decades of exploration, effective treatment strategies for reversing or preventing WMHs are still lacking. Preclinical drugs with great efficacy in experimental models have frequently failed to yield encouraging outcomes, which raise doubts about the translational value of these initial findings. Additionally, the conclusions from clinical trials on whether risk factor management in elderly patients could prevent WMHs progression or ameliorate cognitive impairment are mixing and inconsistent [2, 13].

Limb remote ischemic conditioning (RIC) refers to an approach that brief, repetitive and sublethal ischemia-reperfusion applied to a limb or limbs can induce systemic endogenous tolerance, whereby preventing remote organs or tissues from subsequent (detrimental) ischemic injury [14]. Animal studies have demonstrated that daily limb RIC is effective in reducing WM damage, promoting angiogenesis and improving cognitive function after CCI [15–17]. In a proof-of-concept randomized controlled trial conducted by our study group, we found that chronic RIC was able to alleviate WMHs and slow down cognitive decline in patients with cerebral small-vessel disease (CSVD) [18]. Importantly, regarding the patients with ICAS either above or beneath 80 years of age, chronic RIC is safe and shown to decrease the incidence of stroke recurrence, possibly via enhancing cerebral perfusion and ameliorating plasma biomarkers of coagulation and inflammation [19, 20]. However, whether RIC can alter WMHs in octo- and nonagenarians with ICAS has not been reported yet. Herein, the aim of this study was to investigate whether long-term RIC could prevent the progression of WMHs and improve cognition in octo- and nonagenarians with ICAS.

## RESULTS

### Patient characteristics

A total of 58 patients, including 30 in the RIC group and 28 in the control group, were included in the final analysis. The mean age was 83.5±2.3 years and 84.2±1.6 years for the RIC group and control group, respectively. As shown in Table 1, none of the baseline demographic and clinical characteristics differed significantly between the two groups. There were no statistically significant between-group differences in the Fazekas scale and Scheltens scale scores (Fazekas scores: 3.37±1.03 for RIC vs. 2.93±1.09 for control; Scheltens scores: 14.60±2.79 for RIC vs. 14.86±3.05 for control; all *p*>0.05). The mean MMSE and MoCA scores were similar between groups (MMSE: 24.27±4.65 for RIC vs. 23.82±5.74 for control; MoCA: 22.20±4.42 for RIC vs. 20.89±5.78 for control; all *p*>0.05).

**Table 1:** Baseline characteristics of the 58 patients

Variable	RIC group	Control group	*p*-value
No. of patients	30	28	-
Gender (male/female)	18/12	17/11	0.956
Age, years (mean±SD)	83.5±2.3	84.2±1.6	0.187
Education (junior middle school level of education and above)	29 (96.7)	28 (100.0)	1.000
Smoking	2 (6.7)	3 (10.7)	0.936
Diabetes	13 (43.3)	11 (39.2)	0.754
Hypertension	20 (66.7)	18 (64.3)	0.849
hyperlipidemia	22 (73.3)	20 (71.4)	0.871
Previous stroke	16 (53.3)	16 (57.1)	0.771
Previous TIA	15 (50.0)	15 (53.6)	0.786
Locations of stenosis			
Right ICA	6 (20.0)	5 (17.9)	0.835
Left ICA	4 (13.3)	5 (17.9)	0.910
Right MCA	5 (16.7)	5 (17.9)	1.000
Left MCA	5 (16.7)	4 (14.3)	1.000
BA	2 (6.7)	2 (7.1)	1.000
Multifocal stenosis	8 (26.6)	7 (25.0)	0.885
Medical management			
ACE inhibitors	2 (6.7)	3 (10.7)	0.665
ARBs	3 (10.0)	2 (7.1)	1.000
ARBs plus CCBs	11 (36.7)	10 (35.7)	0.940
CCBs	4 (13.3)	3 (10.7)	1.000
Antidiabetic oral therapy	9 (30.0)	8 (28.6)	1.000
Antidiabetic insulin therapy	4 (13.3)	3 (10.7)	1.000
Anticoagulation and statin therapy before enrollment			
Aspirin	11 (36.7)	12 (42.9)	0.630
Clopidogrel	1 (3.3)	2 (7.1)	0.951
Aspirin plus clopidogrel	0	0	-
Statins	2 (6.7)	3 (10.7)	0.936
Headache (HIT-6) (median IQR)	54 (36–58)	54 (36–56)	0.924
Dizziness	25(83.3)	22 (78.6)	0.644
Sleeping disorder	17 (56.7%)	14 (50%)	0.611
Fazekas scores (mean±SD)	3.37±1.03	2.93±1.09	0.112
Scheltens scores (mean±SD)	14.60±2.79	14.86±3.05	0.702
Overall MMSE scores (mean±SD)	24.27±4.65	23.82±5.74	0.925
Cognition dysfunction evaluated by MMSE*	13 (43.3%)	12 (42.9%)	0.971
Overall MoCA scores (mean±SD)	22.20±4.42	20.89±5.78	0.599
Cognition dysfunction evaluated by MoCA**	25 (83.3%)	25 (89.3%)	0.783
NIHSS scores (mean±SD)	11.3±2.3	11.1±2.6	0.757
mRS scores (mean±SD)	3.4±0.6	3.5±0.5	0.495

Values are given as n. (%) unless otherwise indicated. *Cognition dysfunction was defined as MMSE score<24 in patients with higher than or equal to junior middle school level of education and MMSE score<20 in patients with primary school level of education. **Cognition dysfunction was defined as MoCA<26. Abbreviation: TIA=transient ischemic attack; ICA=internal carotid artery; MCA=middle cerebral artery; BA=basilar artery.

### WMHs evaluation

### *Fazekas scores*

In the RIC group, Fazekas scores were significantly lower than those in the control group at 300-day follow-up (both unadjusted and adjusted *p*<0.001), (Table 2). Compared with the baseline, Fazekas scores in the RIC group were significantly decreased at both 180-day and 300-day follow-ups (*p*<0.05 and *p*<0.01). Moreover, 300-day RIC treatment was effective as 180-day RIC treatment in terms of decreasing the Fazekas scores (*p*>0.05). By contrast, there was no significant difference in the baseline, 180-day and 300-day Fazekas scores in the control group (*p*>0.05).

**Table 2:** WMHs and Cognition evaluation at baseline, 180-day and 300-day

	RIC group	Control group	*p*-value	Adjusted *p*-value
WMHs evaluation				
Fazekas scores				
180-day	2.53±0.63*	2.86±0.97	0.275	0.089
300-day	2.07±0.25**	2.82±0.86	<0.001	<0.001
Scheltens scores				
180-day	12.87±2.33*	15.29±2.84	0.001	<0.001
300-day	11.07±2.15**	16.93±3.42**	<0.001	<0.001
				
Cognition evaluation				
Overall MMSE scores				
180-day	27.10±2.95**	24.68±4.95	0.030	0.006
300-day	27.70±2.48***	24.11±4.86	0.001	<0.001
Overall MoCA scores				
180-day	26.70±2.65***	23.11±5.02***	<0.001	<0.001
300-day	26.80±2.22***	22.71±4.84***	<0.001	<0.001
				
Other clinical symptoms				
HIT-6 scores				
180-day	36 (36–37)	46 (36–48)	<0.001	<0.001
300-day	36 (36–36)	46 (36–48)	<0.001	<0.001
Dizziness, n (%)				
180-day	7 (23.3)	18 (64.3)	0.002	0.001
300-day	5 (16.7)	20 (71.4)	<0.001	<0.001
Sleeping disorder, n (%)				
180-day	9 (30.0)	15 (53.6)	0.069	0.024
300-day	7 (23.3)	16 (57.1)	0.009	0.003

The discrepancies between the two groups were analyzed through Mann-Whitney U test or Chi-square analysis. Post-treatment vs. pretreatment was processed using Friedman test (*p<0.05, **p<0.01, ***p<0.001). Adjust p-value was adjusted for hypertension, diabetes and hyperlipidemia.

### *Scheltens scores*

**Figure 1:**
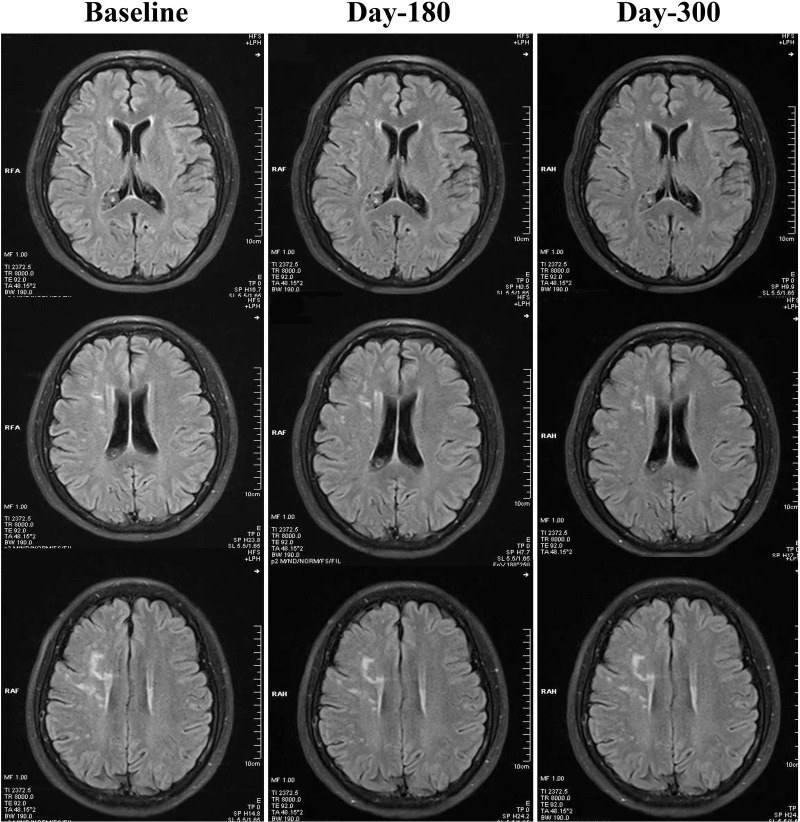
**Changes of WMHs in the RIC group from baseline, day 180 to day 300**. Compared with baseline, WMHs in the RIC group were significantly decreased at both day 180 and day 300 follow-ups.

**Figure 2:**
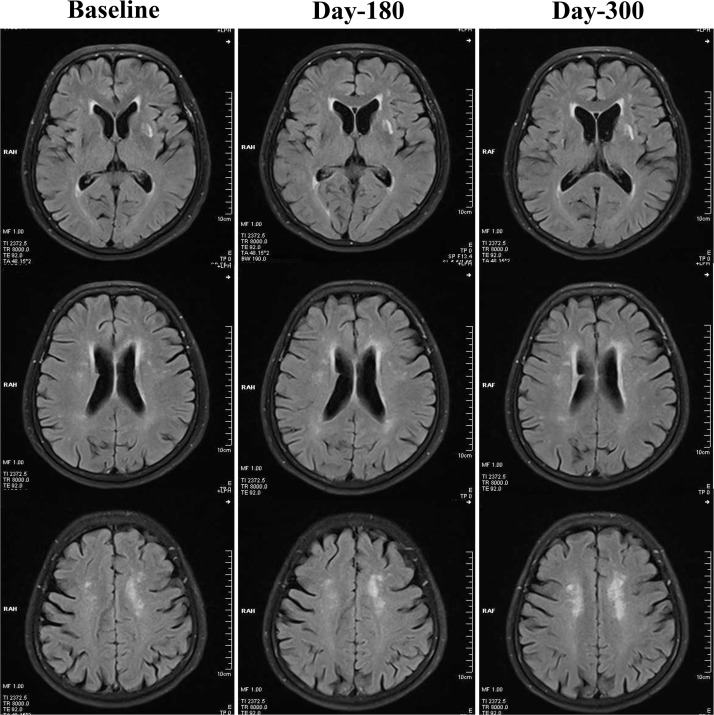
**Changes of WMHs in the control group from baseline, day 180 to day 300**. In the control group, WMHs were not significantly attenuated at day 180 or day 300, when compared with baseline levels. By contrast, WMHs seemed to be increased robustly at day 300.

As demonstrated in Table 2, both 180-day (unadjusted *p*=0.001, adjusted *p*<0.001) and 300-day (both unadjusted and adjusted *p*<0.001) RIC treatments significantly decreased scores on the Scheltens scale, as compared with the control group. We also found a statistically significant reduction in the Scheltens scores of the RIC group with time and over days (180-day vs. baseline,* p*<0.05; 300-day vs. baseline, *p*<0.01), Figure 1. There was no significant difference in the Scheltens score between the baseline and the 180-day follow-up in the control group, but the score was robustly increased at 300-day (180-day vs. baseline,* p*>0.05; 300-day vs. baseline, *p*<0.001), Figure 2.

### Cognition evaluation

### *MMSE scores*

With regards to the overall MMSE score, there was a statistically significant difference favoring RIC at both 180-day (unadjusted *p*=0.03, adjusted *p*=0.006) and 300-day (unadjusted *p*=0.001, adjusted *p*<0.001) follow-ups, Table 2. In addition, there was an increase in the overall MMSE score after 180-day and 300-day RIC treatments in comparison to the baseline level (*p*<0.01 and *p*<0.001), Supplementary Table 1. However, no similar improvement was observed in the control group (all *p*>0.05).

An analysis of MMSE subitems was conducted as well, revealing that patients receiving 180-day RIC treatment had significantly higher delayed recall and calculation subscores (*p*=0.008 and *p*=0.018), while those receiving 300-day RIC treatment displayed robustly higher immediate memory, delayed recall and reading subscores (*p*=0.003, *p*<0.001 and *p*=0.033), apart from improved delayed recall and calculation subscores (*p*<0.001 and *p*=0.002), when compared to those in controls, Supplementary Table 1. The remaining subitems failed to yield any significant group differences (*p*>0.05). Regarding the discrimination between the pre-treatment and post-treatment RIC groups, the delayed recall, calculation and immediate memory domains demonstrated the greatest improvement relative to the other cognitive domains, Supplementary Table 1.

### *MoCA scores*

When measured at day 180 and day 300, we noticed significantly increased overall MoCA scores in the RIC group as compared to the control group (all *p*<0.001), Table 2. Changes in the overall MoCA scores reached statistical significance in both groups from baseline to day 180 or day 300 (both *p*<0.001), Table 2.

Examining by MoCA subitems, patients in the control group displayed robustly lower delayed recall (*p*<0.001), attention (*p*<0.001), language (*p*=0.034) and orientation (*p*=0.019) subscores at 180-day follow up when compared with those in the RIC group, and the beneficial effects provided by RIC were sustained at 300-day follow-up, Supplementary Table 2. By day 180, besides the subscores in the four cognitive domains, visuospatial and executive abilities were also improved significantly in the RIC group (all *p*<0.05), Supplementary Table 2.

### Correlations between WMHs visual rating scales and cognition rating scales

The correlations between WMHs (assessed by the Fazekas scale and Scheltens scale) and cognition (assessed by the MMSE and MoCA-BJ) were analyzed by Spearman correlation analysis. As shown in Table 3, the Fazekas and Scheltens scores were significantly related to the overall MMSE and MoCA scores at baseline, 180-day and 300-day (all* p*<0.001). Moreover, correlation data between the two cognitive scales and between the two WMHs rating scales at different time points also showed strong relevance (all* p*<0.001), Supplementary Table 3.

**Table 3:** Spearman correlation analysis between MMSE/MoCA scores vs. Fazekas/Scheltens scores

Baseline (r, p-value)	MMSE	MoCA
Fazekas scores	-0.607, p<0.001	-0.607, p<0.001
Scheltens scores	-0.548, p<0.001	-0.559, p<0.001
180-day (r, p-value)	MMSE	MoCA
Fazekas scores	-0.583, p<0.001	-0.444, p<0.001
Scheltens scores	-0.555, p<0.001	-0.633, p<0.001
300-day (r, p-value)	MMSE	MoCA
Fazekas scores	-0.667, p<0.001	-0.622, p<0.001
Scheltens scores	-0.665, p<0.001	-0.728, p<0.001

### Clinical symptoms

At both 180-day and 300-day follow-ups, patients in the RIC group showed a significant improvement on the clinical symptoms including headache (Headache Impact Test-6 scores: 180-day, 36 (36–37) vs. 46 (36–48), both unadjusted and adjusted *p*<0.001; 300-day, 36 (36–36) vs. 46 (36–48), both unadjusted and adjusted* p<*0.001), dizziness (180-day, 23.3% vs. 64.3%, unadjusted *p*=0.002 and adjusted *p*=0.001; 300-day, 16.7% vs. 71.4%, both unadjusted and adjusted* p*<0.001) and sleeping disorder (180-day, 30.0% vs. 53.6%, unadjusted *p*=0.069 and adjusted *p*=0.024; 300-day, 23.3% vs. 57.1%, unadjusted *p*=0.009 and adjusted *p*=0.003), compared with those in the control group.

## DISCUSSION

The principal findings from the present randomized clinical trial exploring the effect of RIC on WMHs and cognition in octo- and nonagenarians with ICAS are that long-term twice-daily RIC combined with standard medical therapy is clinically effective in ameliorating WMHs and improving cognitive function when compared with standard medical therapy alone.

WMHs of presumed vascular origin are often found in elderly individuals, particularly those with vascular risk factors or vascular diseases. Recent studies have shown that intracranial large artery stenosis may result in WMHs, high loads of which are believed to be associated with increased risk of cognitive impairment, gait abnormalities, stroke, dementia and some unspecific symptoms such as headache, dizziness and sleep disturbance [6, 8, 10, 20]. Measuring the severity and progression of WMHs has been proposed as a useful surrogate marker for predicting therapeutic outcomes. Despite the lack of the optimal method of measuring WMHs, varied visual rating scales and volumetric measurements based on computerized techniques have been used in clinical and research settings. Unlike semi-automated volumetric methods, visual rating scales are relatively less time-consuming and much easier to perform on the MRI scans obtained from different devices. Moreover, there is ample evidence showing that volumetric and visual rating methods for the measurement of WMHs are moderately or highly correlated, and they may yield nearly equivalent estimations of WMHs burden [21, 22]. Hence, in this study, two widely used rating scales including the Fazekas scale and Scheltens scale were adopted to evaluate WMHs amongst elderly patients with ICAS. Importantly, we found that twice-daily RIC therapy for either 180-day or 300-day significantly reduced WMHs scores compared with controls, and longer-term use of RIC may be more effective than shorter-term treatment (lower Fazekas and Scheltens scores at 300-day than 180-day). In contrast, patients treated with medical therapy alone seemed to have more severe WMHs measured at 300-day follow-up relative to that at the baseline. Our results are consistent with previous studies examining the neuroprotective effects of RIC on experimental animal models, which demonstrated that chronic limb RIC could reverse or attenuate WM damage induced by bilateral carotid artery occlusion-related CCI [15, 17]. Additionally, a more recent pilot study enrolling patients with CSVD showed that daily RIC for 1-year was associated with a substantially lower mean WMHs volume than that in the control group [18]. Although we did not explore the detailed mechanisms underlying the RIC-mediated protective effect on WMHs in this study, such benefits may be largely attributable to improved CBF resulting from cerebral angiogenesis, collateral formation and vascular remodeling [14, 17, 19]. Furthermore, RIC has been shown to reduce inflammatory responses, downregulate microglial expression, inhibit the apoptosis of oligodendrocytes, and attenuate production of free radicals, all of which may facilitate the recovery of WMHs derived from ischemic injury [14–17]. Interestingly, our previous study using the same group of patients demonstrated that RIC administered for 180-day markedly reduced the incidence of stroke or TIA recurrence [27]. Given the association between WMHs and stroke or TIA recurrence, we may speculate that this clinical benefit is, at least partially, due to attenuation of WMHs.

Vascular cognitive impairment comprises a clinical spectrum ranging from mild cognitive decline (MCD) to vascular dementia, which poses a great threat to patients’ quality of life. Therefore, early and routine screening for cognitive deficits with sensitive instruments is required, knowing that it allows for timely initiation of interventions to prevent cognitive deterioration. While the MMSE is the most commonly used screening test for cognitive impairment in the world, the MoCA has been proven to be more sensitive than the MMSE for the detection of MCD and mild dementia [23–25]. In the present study, we also observed different percentages of patients with cognitive impairment at baseline depending on the type of screening test used: RIC group, MMSE (13%) vs. MoCA (25%); control group, MMSE (12%) vs. MoCA (25%). Compared with pretreatment, the post-treatment overall MMSE scores in the RIC group were significantly increased, whereas no such change was observed in the control group. Moreover, the overall MoCA scores measured at both 180-day and 300-day follow-ups were substantially increased in both groups, whereas the increases were more pronounced in the RIC group, implying that RIC may confer extra benefits over currently used medical therapies for improving cognition. This result may be supported by the evidence of experimental studies, which demonstrated that long-term RIC could improve cognition via enhancing spatial and working memory after CCI [15–17]. Notably, the result of MMSE and MoCA subitems showed that patients in the RIC group had significantly higher delayed recall, calculation, attention and orientation subscores. Compared with MMSE, MoCA is acknowledged as a potentially more sensitive method to evaluate cognitive domains including visuospatial and executive functions. In our study, although there was no significant difference in the visuospatial and executive subscores between groups, these domains were improved in the RIC group relative to the baseline levels.

Population-based studies have found an association between WMHs and cognition, knowing that increased WMHs burden can contribute to poorer cognitive function [6, 26]. We also noticed a significant negative correlation between WMHs (assessed by the Fazekas scale and Scheltens scale) and cognition (assessed by the MMSE and MoCA-BJ) at baseline, 180-day and 300-day. Therefore, it is reasonable to infer that improved cognition may be secondary to reduced WMHs and enhanced CBF.

Our study is of high clinical relevance due to the following reasons. First of all, to the best of our knowledge, this is the first randomized controlled clinical trial reporting the efficacy of RIC on WMHs reduction and cognition improvement amongst patients with ICAS-induced CCI. ICAS is considered as the most common cause of both stroke and CCI in Chinese and even whole Asian populations. At present, studies designed to explore approaches for slowing down or even reversing the progression of ICAS-associated WMHs are sparse. Second, given the overwhelming role of age on clinical outcomes, uncertainty exists regarding the benefits and risks related to current therapeutic recommendations applicable to elderly patients, particularly octo- and nonagenarians. Hence, clinicians should be intensely cautious in the prevention and treatment of adverse events at this advanced age with functional deterioration and multimorbidity. Unlike other clinical trials of RIC restricted to relatively younger patients, all participants in our study were in the very elderly age (older than 80 years) and showed good compliance within 300-day follow-up. Moreover, the RIC device used in the study provides many advantages, such as simplicity (can be performed automatically), low cost (can be performed repetitively) and mild or no adverse effect, all of which render it an optimal option for inpatients as well as home-care patients.

One of the major limitations of the present study was the small number of patients enrolled from a single center in China. Consequently, this study should be viewed as exploratory and cannot be generalized to the overall or other ethnic populations at this stage. Another limitation was that the follow-up period was not long enough, given the progressive feature of WMHs and cognitive function in the elderly patients. In addition, we only used two visual rating scales to assess the severity of WMHs and did not analyze the microstructural alterations within visible lesions as well as in the surrounding normal-appearing WM. The underlying mechanisms of RIC-mediated neuroprotection, such as WMHs reduction, and cognition or symptoms improvement were not fully investigated. Last but not least, we applied a previously reported RIC protocol in our patients, bur whether modifying RIC protocol in this clinical setting could achieve better outcomes remains unknown. A series of larger prospective clinical trials (Clinical Trials.gov: NCT03105141 and NCT02534545) is ongoing in our study group in an attempt to work out ways to tackle these challenges.

## MATERIALS AND METHODS

### Study design and participants

This study was a single center, prospective and randomized controlled clinical trial. The study protocol was approved by the Institutional Review Board of Capital Medical University (Beijing, China), and all patients provided written informed consent prior to enrollment. The study design, patient selection and randomization, and part of results have been previously described, (NCT01570231) [27].

Patients were enrolled into the study based on the following criteria: 1) presenting with neurologic symptoms corresponded with the territory of offending vessels; 2) significant stenosis of intracranial arteries (at least one artery stenosed ≥70%) measured by magnetic resonance angiography (MRA) or computed tomography angiography (CTA); 3) age of 80 years or older; 4) TOAST (Trial of Org 10172 in Acute Stroke Treatment) subtype 1, large-artery atherosclerosis; 5) National Institutes of Health Stroke Scale (NIHSS) score less than 15, and modified Rankin Scale (mRS) score of 2 to 4.

Patients were excluded if they met any of the following criteria: 1) presence of significant extracranial arterial stenosis (≥50%); 2) ICAS secondary to non-atherosclerotic etiologies, such as moyamoya disease, artery dissection, intracranial granulomatous arteritis, and any known vasculitic diseases; 3) Intracranial abnormalities such as tumor, vascular malformation, and cerebral venous sinus thrombosis; 4) Intracranial bleeding within 6 months prior to inclusion; 5) Refractory hypertension (systolic blood pressure>200 mmHg) that cannot be controlled by medical intervention; 6) severe renal, hepatic or cardiovascular disorder; 7) Contraindications for RIC, such as soft tissue or vascular injury, fracture and other diseases affecting the upper extremities, and severe subclavian artery stenosis; 8) absence of available MR images identifying WMHs; 9) Lost to follow-up.

### Randomization and procedures

Patients were randomly allocated into two groups in a 1:1 ratio: RIC group and control group, using a computer-generated technique with random blocks. Patients or their guardians performing RIC were not blinded to treatment assignment. However, researchers who participated in the data analysis were masked to the assignment.

Patients in the RIC group received bilateral upper arm ischemic conditioning treatment (patent number ZL200820123637.X, China) twice daily for 300 consecutive days. The RIC protocol consisted of 5 cycles of alternating 5-min upper arm ischemia and 5-min reperfusion, at a cuffing pressure of 200 mmHg, enabling a complete blockage of the arterial and venous blood flow through the forearm simultaneously. In the control group, patients underwent the similar procedure, with the exception of the inflating pressure, which was set as 30 mmHg, to maintain the pressure feeling of patients without any effect on the blood flow (Supplementary Figure 1). Standard medical therapy and vascular risk factors were controlled similarly in the two groups.

### Brain MRI and assessment of WMHs

Brain MRI was performed on a 3.0T scanner system (Siemens, Verio, Germany) at baseline, at 180-day follow-up and at 300-day follow-up. The imaging protocol was mainly comprised of T1-weighted (T1W), T2-weighted (T2W), T2 fluid-attenuated inversion recovery (T2 FLAIR), T2*-weighted gradient recalled echo (GRE), and diffusion-weighted imaging (DWI) sequences, which were all completed with 5-mm thick slices.

WMHs were defined as punctuate and/or diffuse hyperintense lesions on T2W and T2 FLAIR images, but were not seen as apparent hypointense changes on T1W.

Two visual rating scales including the Fazekas scale and Scheltens scale were used to semiquantitatively assess WMHs on the MRI, primarily T2 FLAIR scans [28, 29]. In the Fazekas scale, periventricular and deep WM hyperintensities were scored separately. A total Fazekas score ranging from 0 to 6 were used for statistical comparison by summing the two separate scores. The Scheltens scale has a range from 0–84, where scores 0–6 can be given in the periventricular regions, 0–24 in the deep WM, 0–30 in the basal ganglia, and 0–24 in the infratentorial regions. Two senior neuroradiologists, who were blinded to patients’ clinical data and vascular conditions, performed all the ratings on baseline and follow-up images in a side-by-side fashion. If inconsistency occurred between the two raters, a third rater would be introduced to re-examine WMHs, and the final decision was made based upon the ratings which have the majority, at least two thirds of the votes.

### Assessment of cognitive function

Two screening instruments were used to assess the cognitive function of enrolled patients at baseline and at 180-day as well as 300-day follow-ups, including the Mini-Mental State Examination (MMSE) and Beijing version of the Montreal Cognitive Assessment (MoCA-BJ). Neurologists in our institution who were blinded to the study design and randomization completed all the assessments.

The MMSE is a 30-point questionnaire, which covers the domains of orientation, memory (immediate and delayed recall), calculation, language (naming, execution, repetition, reading and writing) and visuospatial ability [30]. A cutoff score of <24 was chosen to indicate cognitive impairment in patients with higher than or equal to junior middle school level of education [30].

The MoCA-BJ is the most commonly used version of MoCA in Mainland China with a maximal score of 30 [31]. The examination assesses the following cognitive domains: visuospatial and executive skills, naming, short-term memory, attention, memory, abstraction and orientation. Cognitive impairment on the MoCA was defined as a score <26 [31].

### Outcomes

We measured the change of WMHs on T2 FLAIR sequences, estimated by the Fazekas scale and Scheltens scale (significant level α was defined as 0.25 for each scale), the alterations of cognition assessed by the MMSE and MoCA, and clinical symptoms such as headache, dizziness and sleeping disorder, at baseline and at 180-day and 300-day follow-ups.

### Statistical analysis

Continuous variables were described as mean ± standard deviation (SD) or otherwise as median (interquartile range, IQR). Categorical variables were presented as counts and percents. We used the t test or Mann-Whitney U test to compare continuous variables, and the Pearson χ2 test or Fisher’s exact test to compare categorical variables between the two groups. Furthermore, multivariate analyses such as logistic regression model and linear regression model were used to balance the covariates (including hypertension, diabetes and hyperlipidemia). Intragroup comparisons of mean or median values between baseline and follow-up evaluation were conducted by means of the Friedman test in order to remove the bias derived from repeated measurement. The associations between WMHs (measured by the Fazekas scale and Scheltens scale) and cognition (measured by the MMSE and MoCA-BJ) were evaluated by Spearman correlation analysis. A *p* value < 0.05 was considered statistically significant. All statistical analyses were done with SPSS version 21 (SPSS Inc., Chicago, IL, USA).

### Data availability

Anonymized data are available to any qualified investigator for the purpose of reproducing the results or replicating the procedure.

## CONCLUSIONS

In summary, our current findings underline that RIC may prove to be an adjunctive to standard medical therapy for preventing the progression of WMHs and ameliorating cognitive impairment in very elderly patients with ICAS-induced CCI. The clinical implications of this pilot study need to be further explored in future multicenter randomized clinical trials powered for the clinical outcomes.

## SUPPLEMENTARY MATERIAL

Supplementary Figure and Tables
