# Supplementary material for: Efficacy of remote ischemic conditioning on improving WMHs and cognition in very elderly patients with intracranial atherosclerotic stenosis
Source: Aging (Albany NY). 2019 Jan 28;11(2):634–48. doi: 10.18632/aging.101764 (PMC6366980; doi:10.18632/aging.101764)
Supplement: Supplementary Figure and Tables [file aging-11-101764-s001.pdf]

SUPPLEMENTARY MATERIAL

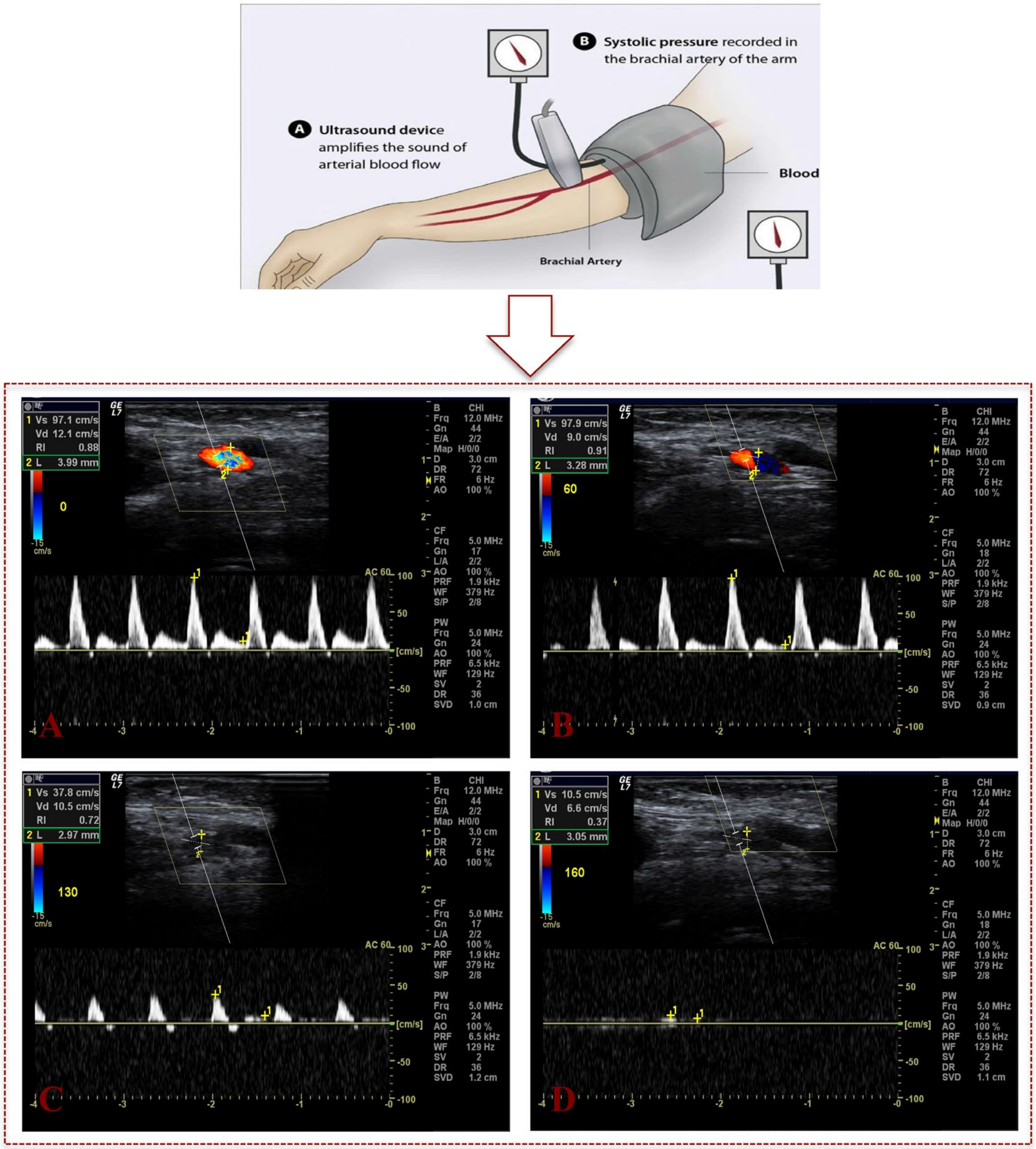

Supplementary Figure 1. The Doppler ultrasound employed in a patient undergoing RIC treatment with different degrees of cuffing pressure. (**A**) When the cuffing pressure is set as 0mmHg, the resistance index (RI) is 0.88 and the arterial and venous blood echo could be seen obviously; (**B**) When the cuffing pressure is set as 60mmHg, the RI is 0.91 and the blood echo is weakened; (**C**) When the cuffing pressure is set as 130mmHg, the RI is 0.72 and the blood echo is nearly diminished; (**D**) When the cuffing pressure is set as 160mmHg, the RI is 0.37 and the blood echo completely disappeared.

Supplementary Table 1. The analysis of MMSE subitems at day 180 and day 300.

	180-day			300-day		
Items (score)	RIC group	Control group	p-value	RIC group	Control group	p-value
Orientation (10)	9.47±0.78	9.07±1.15	0.274	9.53±0.63	8.89±1.13	0.029
Immediate memory (3)	2.83±0.38*	2.57±0.69	0.131	2.93±0.25*	2.54±0.54	0.003
Delayed recall (3)	2.53±0.63*	1.96±0.92	0.008	2.67±0.48**	1.89±0.79	<0.001
Calculation (5)	3.77±0.97*	2.93±1.54	0.018	4.00±0.91***	2.96±1.40	0.002
Naming ability (2)	2.00±0.00	1.96±0.19	0.301	2.00±0.26	1.93±0.26	0.304
Retelling (1)	0.93±0.25	0.86±0.36	0.345	0.97±0.18	0.82±0.39	0.072
Reading (1)	1.00±0.00	1.00±0.00	1.000	1.00±0.00	0.86±0.36	0.033
Execution (3)	2.67±0.71	2.68±0.72	0.873	2.73±0.58	2.68±0.77	0.927
Writing (1)	0.93±0.25	0.82±0.39	0.195	0.93±0.25	0.79±0.42	0.106
Visuospatial ability (1)	0.97±0.18	0.82±0.39	0.072	0.93±0.25	0.75±0.44	0.056
Total scores	27.10±2.95**	24.68±4.95	0.030	27.70±2.48***	24.11±4.86	0.001
Cognition dysfunction evaluated by MMSE†	2 (6.7%)	9 (32.1%)	0.013	1 (3.3%)	9 (32.1%)	0.011

The overall scores and subscores of the MMSE in the groups were presented as median (IQR) or mean ± standard deviation and analyzed using t test or Mann-Whitney U test. Comparisons between post-treatment and pretreatment were processed using Friedman test (*p<0.05, **p<0.01, ***p<0.001). †Cognition dysfunction was defined as MMSE score<24 in patients with higher than or equal to junior middle school level of education and MMSE score <20 in patients with primary school level of education.

Supplementary Table 2. The analysis of MoCA subitems at day 180 and day 300.

	180-day			300-day		
Items (score)	BAIPC group	Control group	p-value	BAIPC group	Control group	p-value
Visuospatial and execution ability (5)	4.33±0.76**	4.11±1.07	0.619	4.33±0.80**	3.93±1.05	0.142
Naming (3)	2.90±0.31	2.71±0.66	0.330	2.83±0.38	2.64±0.68	0.348
Delayed recall (5)	3.90±0.71***	2.96±0.69**	<0.001	4.03±0.62***	3.07±0.77**	<0.001
Attention (6)	5.27±0.58***	4.18±1.19**	<0.001	5.27±0.58***	4.18±1.12**	<0.001
Language (3)	2.87±0.35*	2.50±0.75	0.034	2.83±0.38*	2.36±0.73	0.005
Abstraction (2)	1.93±0.25	1.71±0.66	0.169	1.97±0.18	1.64±0.68	0.016
Orientation (6)	5.50±1.01**	4.93±1.30	0.019	5.53±0.78**	4.89±1.23	0.018
Total score	26.70±2.65***	23.11±5.02***	<0.001	26.80±2.22***	22.71±4.84***	<0.001
Cognition dysfunction evaluated by MoCA††	4 (13.3%)	19 (67.9%)	<0.001	4 (13.3%)	21 (75.0%)	<0.001

The overall scores and subscores of the MoCA in the groups were presented as median (IQR) or mean ± standard deviation and analyzed using t test or Mann-Whitney U test. Comparisons between post-treatment and pretreatment were processed using Friedman test (*p<0.05, **p<0.01, ***p<0.001). ††Cognition dysfunction was defined as MoCA<26 in patients.

Supplementary Table 3. Spearman correlation analysis between MMSE and MoCA scales vs. between Fazekas and Scheltens scales.

	MMSE and MoCA	Fazekas scores and Scheltens scores
Baseline (r, *p*-value)	0.901, $p<0.001$	0.619, $p<0.001$
180-day (r, *p*-value)	0.878, $p<0.001$	0.622, $p<0.001$
300-day (r, *p*-value)	0.898, $p<0.001$	0.795, $p<0.001$
